# Inappropriate Diet Exacerbates Metabolic Dysfunction-Associated Steatotic Liver Disease via Abdominal Obesity

**DOI:** 10.3390/nu16234208

**Published:** 2024-12-05

**Authors:** Minghui Xiang, Xiaoli Tian, Hui Wang, Ping Gan, Qian Zhang

**Affiliations:** 1 National Institute for Nutrition and Health, Chinese Center for Disease Control and Prevention, Beijing 100050, China; alicexmh@163.com; 2 School of Public Health, Xinjiang Medical College, Ürümqi 830000, China; txl1345465854@163.com; 3 School of Public Health, Xinjiang Second Medical College, Karamay 834000, China; 4 Department of Maternal and Child Health, School of Public Health, Peking University, Beijing 100191, China; huiwang@bjmu.edu.cn; 5 Guangdong Provincial Center for Disease Control and Prevention, Guangzhou 511400, China

**Keywords:** metabolic dysfunction-associated steatotic liver disease (MASLD), abdominal obesity, free fatty acids (FFAs), diet, visceral fat

## Abstract

Metabolic dysfunction-associated steatotic liver disease (MASLD) represents a refined categorization of non-alcoholic fatty liver disease (NAFLD), highlighting the intricate relationship between hepatic steatosis and metabolic dysfunction. Abdominal obesity (AO), a key diagnostic criterion for metabolic dysfunction, predominantly results from inappropriate diet and unhealthy dietary habits. To comprehensively investigate which dietary factors contribute to MASLD through AO and to understand the underlying biological mechanisms, we initially conducted a systematic review of meta-analysis articles in the PubMed database from the past decade, summarizing dietary factors that affect AO. Subsequently, we conducted targeted searches in the PubMed database for these dietary factors and provided a narrative review of the mechanisms of how these dietary factors lead to AO and how AO exacerbates MASLD. A diet characterized by excessive intake of energy, carbohydrates, fructose, or ultra-processed foods (UPFs) is considered inappropriate. Inappropriate diet leads to the formation of MASLD and AO by enhancing pathways such as de novo lipid synthesis (DNL) in the liver, insulin resistance (IR), gut–liver dysfunction, and inflammation. Dietary interventions for inappropriate diets can effectively intervene in and improve MASLD and AO. The mechanism of inappropriate diet on abdominal fat deposition is through excessive energy or the activation of the enzyme 11β-hydroxysteroid dehydrogenase type 1 (11β-HSD-1) to increase endocortisol secretion. Then, the excessive accumulation of visceral fat facilitates a rapid and augmented flux of free fatty acids (FFAs) to the liver and initiates a series of deleterious effects, including oxidative stress (OS), endoplasmic reticulum stress (ERS), activation of protein kinase C (PKC) pathways, and inflammation. Additionally, FFAs may mediate excessive lipid deposition and hepatocellular damage through the action of hormones. These pathways to liver damage exacerbate MASLD and progression to metabolic dysfunction-associated steatohepatitis (MASH) and fibrosis. Furthermore, investigating other potential mechanisms by which AO may influence MASLD could offer new recommendations for the treatment guidelines of MASLD.

## 1. Introduction

In 2020, the concept of metabolic dysfunction-associated steatotic liver disease (MASLD) was introduced to distinguish it from non-alcoholic fatty liver disease (NAFLD) [1]. For a diagnosis of MASLD, an individual must exhibit at least one specific cardiometabolic criterion—BMI, waist circumference, blood pressure, fast serum glucose, plasma triglycerides, and plasma high-density lipoprotein cholesterol—alongside evidence of hepatic steatosis [1,2], with the exclusion of cardiometabolic criteria. MASLD stands out as one of the emerging global health challenges [3]. If left untreated, patients with MASLD can experience progressive hepatic deterioration, culminating in steatohepatitis; liver fibrosis; hepatocellular carcinoma; and eventually, fatal liver failure [3,4]. Between 1990 and 2021, the global prevalence of MASLD surged from 1.69 million cases (95% uncertainty interval [UI], 1.29–2.21 million disability-adjusted life years [DALYs]) to 3.67 million cases (95% UI, 2.90–4.61 million DALYs), marking a 2.2-fold increase in affected individuals and a substantial escalation in the associated health burden [5]. The introduction of MASLD has also underscored the close association between fatty liver diseases and metabolic dysfunction. A 2021 meta-analysis of NAFLD prevalence among 1,201,807 individuals globally indicated an incidence rate of approximately 8 cases per 100,000 people annually, or 14,133.0 (95% confidence interval [CI], 2765.2–25,500.7), which is notably higher than the prevalence in overweight/obese populations, estimated at 416.6 cases per 100,000 people (95% CI, 5687.3–11,145.8) [6].

Abdominal obesity (AO) is a significant contributor to the development of fatty liver disease, defined by an excess waist circumference (WC). AO is a hallmark of metabolic syndrome and is closely linked to the onset of fatty liver disease, exhibiting a high prevalence in the population. A cross-sectional study conducted among 7238 adults in North China from 2013 to 2016 demonstrated a significant correlation between adult waist circumference and visceral obesity index with the prevalence of NAFLD [7]. Data from the National Health and Nutrition Examination Survey (NHANES) in the United States indicated that, in 2018, approximately 53.13% of American adults had AO, surpassing the proportion of the general obese population (35.48%) [8]. According to the China Nutrition and Health Surveys, the prevalence of AO among adults was 29.3% in 2015, higher than the obesity rate of 14.1%. Additionally, the report noted that from 2016 to 2017, 14.4% of children and adolescents aged 7 to 17 years exhibited AO, exceeding the overweight and obesity rate of 11.1% in this age group [9].

Previous population studies have identified a significant association between excessive intake of saturated fatty acids, short-chain fatty acids, carbohydrates, added sugars, and UPFs (inappropriate diet) and MASLD [10]. The established pathological mechanisms include the following: high carbohydrate intake primarily leads to lipid deposition in hepatocytes through the de novo lipogenesis pathway in the liver [11]; excessive consumption of added sugars and short-chain fatty acids results in hepatic lipid deposition through gut metabolism [12]. Furthermore, research has shown that dietary fiber can improve the distribution and abundance of gut microbiota, thereby reducing hepatic lipid deposition and maintaining metabolic balance by preserving gut barrier function, enhancing immune function, and increasing the metabolism of folate and unsaturated fatty acids [13].

AO, as an indicator of metabolic syndrome and one of the risk factors for MASLD, is also closely associated with inappropriate dietary intake. However, no comprehensive discussion has been published on the relationship between diet, AO, and MASLD. Therefore, this paper reviews the dietary factors contributing to AO and the mechanisms by which inappropriate diet exacerbates MASLD through AO. It clarifies the importance of AO as a risk and warning indicator for MASLD and provides a comprehensive theoretical framework for the dietary intervention and treatment of MASLD.

In this review, we integrated both systematic and narrative approaches to synthesize the evidence to gain a comprehensive understanding of the interplay and underlying molecular mechanisms between diet, AO, and MASLD. We initially conducted a systematic search, collation, and screening of meta-analyses published on PubMed over the past decade. These focused on dietary patterns, foods, and nutrients that can alter WC or other AO indicators. By integrating these findings with other relevant research articles, we analyzed which diets are appropriate for improving AO or reducing WC and which may increase the risk of AO. Subsequently, we extensively reviewed clinical and murine experimental studies to summarize and elucidate the molecular mechanisms by which specific foods or nutrients contribute to AO and how this condition exacerbates MASLD. Through these steps, we provide a complete overview of how inappropriate diets can exacerbate MASLD through AO, offering a theoretical basis for dietary interventions in MASLD and the use of AO as an early warning factor for MASLD.

## 2. Diet and Abdominal Obesity

Dietary intake is a significant contributor to AO, with high-energy diets and excessive consumption of specific nutrients, such as fructose, playing a pivotal role. A systematic search and analysis of meta-reviews related to WC, AO, foods, nutrition, and dietary patterns published on PubMed over the past decade yielded the following findings (Supplementary S1). The World Health Organization (WHO) employs WC as a criterion for assessing AO in adults. However, due to variations in fat distribution and ethnicity, the standards for AO differ among countries. In health status analysis studies of European populations, such as in the United Kingdom, adults are considered to have AO if the WC is ≥102 cm for males and ≥88 cm for females [14]. In contrast, the standards for AO in Asian countries, exemplified by China, are more stringent, with the threshold set at ≥90 cm for adult males and ≥80 cm for adult females [15]. Therefore, in the following study, WC was analyzed both as a continuous variable and as a categorical variable based on whether it met the AO criteria, and its association with dietary factors was examined.

### 2.1. Dietary Patterns for Improving Waist Circumference

**Figure 1:**
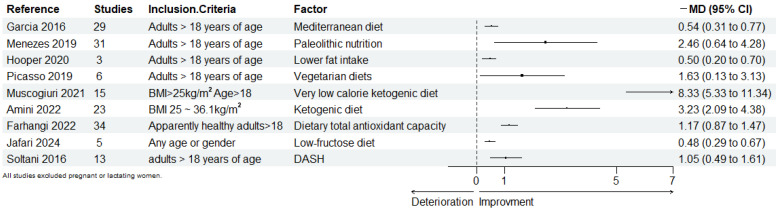
Forest map of dietary patterns for improving waist circumference. The size of the points is proportional to the standard error. DASH: Dietary Approaches to Stop Hypertension [16,17,18,19,20,21,23,24,25].

Within the category of dietary patterns (Table 1), several meta-reviews have been shown to significantly improve WC in both healthy and unhealthy adults (Figure 1): the Mediterranean diet groups versus non-Mediterranean diets and control groups prior to the Mediterranean diet intervention (mean difference [MD]: −0.54 cm, 95% confidence interval [CI] −0.77 to −0.31 cm) [16]; the Paleolithic diet groups versus control groups prior to Paleolithic diet intervention (MD: −2.46 cm, 95% CI −4.28 to −0.64 cm) [17]; the lower-fat diet versus higher fat diet (MD: −0.5 cm, 95% CI −0.7 to −0.2 cm) [18]; higher categories of total antioxidant capacity (TAC) groups versus lower categories of TAC groups (MD: −1.17 cm, 95% CI −1.47 to −0.87 cm) [19]; and vegetarian diet groups compare with omnivore diet groups (MD: −1.63 cm, 95% CI −3.13 to −0.13 cm) [20]. Additionally, a meta-comparison of a low-fructose diet (LFD) and a regular diet (MD: −0.48 cm, 95% CI −0.67 to −0.29 cm) included studies with both children and adults, demonstrating significant improvements in WC across all age groups with LFD [21]. However, the Mediterranean diet’s effect on WC in children was not significant when compared to both the standard diet and the low-fat diet control groups (MD: −0.12, 95% CI −0.29 to 0.06 cm) [22]. The very-low-calorie ketogenic diet (MD: −8.33 cm, 95% CI −11.34 to −5.33 cm), ketogenic diets (MD: −3.23 cm, 95% CI −4.38 to −2.09 cm), and Dietary Approaches to Stop Hypertension (DASH) versus other diets in studies (MD = −1.05 cm, 95%CI −1.61 to −0.49 cm) have been shown to significantly reduce WC in adults with overweight and obesity [23,24,25].

### 2.2. Foods for Improving Waist Circumference

**Figure 2:**
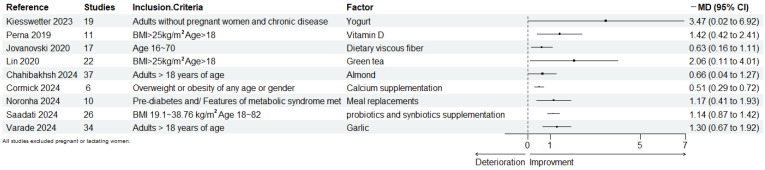
Forest map of foods for improving waist circumference. The size of the points is proportional to the standard error [26,27,28,29,30,31,32,33,34].

Among various foods and food components, several meta-reviews have been found to significantly improve WC in both healthy and unhealthy adults (Figure 2): Viscous fiber-rich foods (MD: −0.63 cm, 95% CI −1.11 to −0.16 cm) [26] and meal replacements (MD: −1.17 cm, 95% CI −1.93 to −0.41 cm) [27] may improve AO by enhancing satiety and reducing caloric intake. Almonds (MD: −0.66 cm, 95% CI −1.27 to −0.04 cm) [28], garlic (MD: −1.30 cm, 95% CI −1.92 to −0.67 cm) [29], and green tea (MD: −2.06 cm, 95% CI −4.01 to −0.11 cm) [30] may ameliorate AO through enhanced fat oxidation and anti-inflammatory effects. Yogurt (MD: −3.47 cm, 95% CI −6.92 to −0.02 cm) [31], probiotics, and synbiotics (MD: −1.14 cm, 95% CI −1.42 to −0.87 cm) [32] may improve AO by modulating gut microbiota. Additionally, vitamin D (MD: −1.42 cm, 95% CI −2.41 to −0.42 cm) [33] and calcium intake (MD: −0.51 cm, 95% CI −0.72 to −0.29 cm) [34] may also contribute to the improvement in AO by enhancing the functionality of adipocytes.

### 2.3. Diets Associated with Abdominal Obesity and General Obesity

**Figure 3:**
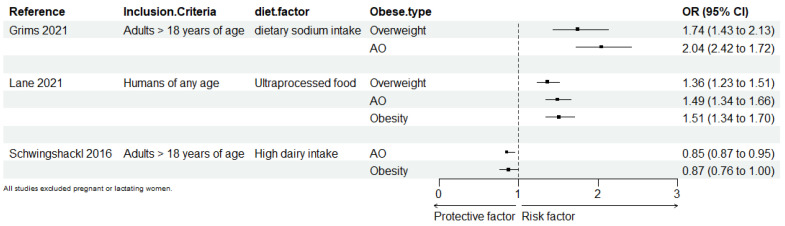
Forest map of diets associated with abdominal obesity and general obesity. The size of the points is proportional to the standard error. AO: abdominal obesity [35,36,38].

Several meta-analyses have been conducted to evaluate the strength of the associations between various foods and food components and the risks of general obesity and AO (Figure 3). Key findings from these studies reveal that high salt intake and the elevated sodium content in UPFs can adversely affect fat metabolism, thereby contributing to obesity. Specifically, a high dietary sodium intake has been linked to an increased risk of AO (OR = 2.04; 95% CI: 1.72, 2.42) and general obesity (OR = 1.74; 95% CI: 1.43, 2.13) in adult populations [35]. Similarly, the consumption of UPFs has been shown to elevate the risk of AO (OR = 1.41; 95% CI: 1.18, 1.68), overweight status (OR = 1.36; 95% CI: 1.14, 1.63), and general obesity (OR = 1.55; 95% CI: 1.36, 1.77) [36,37].

Conversely, the consumption of dairy products appears to have a beneficial effect on fat metabolism, potentially reducing the risk of AO. Studies indicate that higher dairy intake is associated with a reduced risk of AO (OR = 0.85; 95% CI: 0.76, 0.95) and general obesity (OR = 0.87; 95% CI: 0.76, 1.00) [38].

Additionally, meta-analyses have found that increased grain consumption is associated with weight reduction in adults but does not affect WC [39]. Various studies have also identified different impacts of specific food factors on the risk of AO versus general obesity: a cross-sectional study conducted in Sichuan, China, from 2018 to 2019, involving 40,877 adults aged 30 to 79 years, found that consuming spicy foods is a risk factor for AO [40]; a cohort study carried out in rural Henan, China, from 2015 to 2017, involving 28,773 individuals, also demonstrated a significant positive correlation between the frequency of spicy food consumption and the risk of AO [41]. However, other research suggests that spicy foods may have a protective effect against the risk of general obesity [42].

These findings highlight the complex and sometimes contradictory effects of dietary factors on different types of obesity, emphasizing the need for further research to clarify these relationships and inform more nuanced dietary guidelines.

## 3. Mechanisms of Inappropriate Diet Contributing to Abdominal Obesity

AO is primarily characterized by the excessive accumulation of fat tissue within the abdominal cavity, often referred to as visceral obesity. Excessive visceral fat is a key phenotype of visceral obesity. Inappropriate dietary habits (excessive intake of energy, carbohydrates, fructose, or UPFs) can accelerate the deposition of visceral fat, leading to AO.

### 3.1. Characteristics of Visceral Fat

Visceral fat is distributed around various organs within the abdominal cavity, providing protection and support for the viscera (liver, pancreas, intestines, etc.), accounting for 10–20% of total body fat in males and 5–8% in females [43,44]. In addition to the differences in distribution within the body, there are also functional disparities between visceral and subcutaneous fat, with the former posing a greater risk for disease [45,46].

Visceral fat exhibits higher metabolic activity compared to subcutaneous fat, facilitating the storage and consumption of lipids more readily [47]. It is more sensitive to neural signals and cortisol than subcutaneous fat [47]. Visceral fat has the unique ability to secrete inflammatory factors, unlike subcutaneous fat. The adipocytes within visceral fat exhibit lower sensitivity to insulin compared to those in subcutaneous fat [47]. The fatty acids released from the breakdown of visceral adipose tissue (VAT) are primarily drained directly to the liver via portal vein circulation, whereas those from subcutaneous adipose tissue (SCAT) are released into systemic venous circulation [47,48]. Visceral fat contains a higher proportion of inflammatory and immune cells, with a lower capacity for pre-adipocyte differentiation and a higher percentage of large adipocytes [47,48]. Consequently, an excess of visceral fat can cause greater metabolic damage to organs such as the liver compared to an excess of subcutaneous fat [43].

### 3.2. The Mechanism of Inappropriate Diet Influencing Visceral Fat

In addition to excessive caloric intake leading to an exacerbation of abdominal fat deposition, current research on the differential effects of diet on subcutaneous and visceral fat in human populations is scarce. However, mechanistic studies based on cellular and animal experiments have revealed that excessive fructose intake can lead to the formation of MASLD and lipid accumulation in visceral fat cells [49,50]. This occurs through the overexpression of hexose-6-phosphate dehydrogenase (H6PD) within the endoplasmic reticulum lumen and increased cellular inflammation, which together activate 11β-HSD-1 [51]. This activation results in the local production of active cortisol within visceral adipose tissue. The increased concentration of cortisol in visceral fat accelerates the excessive lipid accumulation and lipotoxicity in visceral adipocytes [52]. Furthermore, the activation of 11β-HSD-1 in visceral fat cells may also contribute to the development of insulin resistance [50].

Research on the mechanisms by which various diets influence visceral fat cell lipid deposition signaling pathways, independently of subcutaneous adipose tissue, is relatively scarce. However, studies utilizing obese mouse models have revealed that fructose, high-carbohydrate diets, UPF, and foods rich in saturated fatty acids can promote inflammation in the gut and other organs during metabolism, thereby activating 11β-HSD-1 and facilitating the ectopic deposition of visceral fat [53,54,55,56,57,58,59]. On the other hand, dietary fiber, anti-inflammatory foods (such as tea), fermented foods, and supplementation with diverse gut bacteria can improve the homeostasis and diversity of the gut microbiota, thereby ameliorating the deposition of visceral fat [60,61,62,63].

## 4. Mechanism of Abdominal Obesity Exacerbating MASLD

Excessive visceral fat can lead to the formation of fatty liver disease (FLD) [64]. The liver, as a crucial organ for systemic fat metabolism, is capable of taking up excess free fatty acids (FFAs) and synthesizing them into triglycerides, which are then stored within the cytoplasm in the form of lipid droplets [65]. When there is an overabundance of lipid intake and adipose tissue reaches its capacity to store additional energy, lipolysis in adipose tissue accelerates, reducing the uptake of fatty acids and consequently increasing the concentration of FFAs in the body [64,66]. In response, hepatocytes partially compensate for the function of adipocytes by synthesizing more triglycerides that accumulate in the liver [65].

The accumulation of excessive FFAs, triglycerides, and their metabolic byproducts may exert direct cytotoxic effects on hepatocytes. Within the cytoplasm, these substances exacerbate the development of MASLD and induce insulin resistance through various mechanisms.

### 4.1. Oxidative Stress and Endoplasmic Reticulum Stress

**Figure 4:**
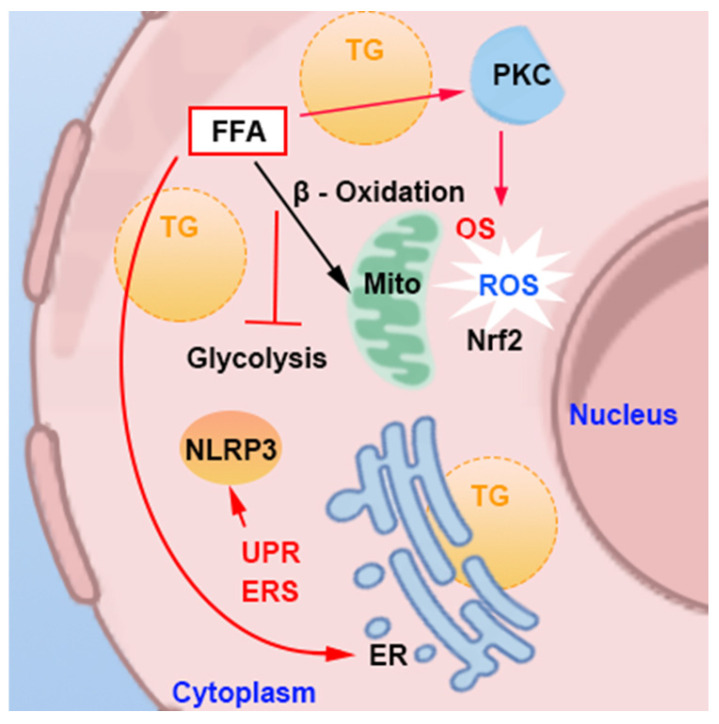
Mechanisms of excess free fatty acids cause oxidative stress and endoplasmic reticulum stress in hepatocyte. FFA: free fatty acid. TG: triglyceride. PKC: protein kinase C. OS: oxidative stress. ROS: reactive oxygen species. ER: endoplasmic reticulum. ERS: endoplasmic reticulum stress. UPR: unfolded protein response. Mito: mitochondria. Red arrows: activate; black arrows: metabolic pathway.

Abdominal obesity-induced lipid deposition and increased levels of FFA can harm hepatocytes via pathways involving OS and ERS (Figure 4), as well as through the interaction between these two pathways, promoting hepatocyte degeneration and apoptosis.

Excess FFAs undergo β-oxidation in the liver, generating acetyl-CoA, which enters the tricarboxylic acid (TCA) cycle. Simultaneously, fatty acid oxidation primarily occurs through mitochondrial oxidation and NADPH oxidase (NOX) enzyme oxidation, both of which produce substantial amounts of reactive oxygen species (ROS). The resulting peroxides can cause mitochondrial damage in hepatocytes, impairing their ability to produce sufficient ATP for cellular needs, leading to dysregulation of lipid metabolism, glucose intolerance, and disturbances in protein synthesis [67]. Additionally, this OS and ERS contribute to hepatocyte injury through apoptotic and ERS signaling pathways. When the TCA cycle cannot consume the excess FFAs, hepatocytes will reutilize acetyl-CoA to synthesize triglycerides, which are stored in the liver, causing lipid accumulation. Studies have observed competition for oxidation substrates between FFA and glucose in the myocardium and diaphragm muscles of rats, where an increase in FFA oxidation leads to decreased glucose oxidation, resulting in glucose accumulation and insulin resistance [68]. However, when researchers inhibited fatty acid oxidation for energy production in the liver and induced excess FFA, insulin resistance was still present [69]. Further investigation revealed that FFA accumulation directly promotes the translocation of protein kinase C (PKC) isoforms from the cell membrane to the cytoplasmic membrane, enhancing oxidative activity, and damages insulin signaling through the FFA-PKC δ-NADPH oxidase and OS-IKKβ/JNK signaling pathways [68,70].

In patients with NAFLD, the activation of the unfolded protein response (UPR) has been identified, triggering the activation of three primary ERS pathways—PERK, ATF6, and IRE1—aimed at alleviating UPR damage [71]. The activation of these ERS signaling pathways increases hepatocyte apoptosis [71,72,73]. As FFA concentration rises, the level of ERS also increases, accelerating the progression of steatohepatitis [73].

There exists an interplay between oxidative reactions and ERS: the activation of antioxidant mechanisms in hepatocytes, such as the Nrf2-ARE pathway, can exacerbate ERS [74,75]. The activation of ATF6 in the ERS response pathway leads to increased CHOP production, inducing cell death and inflammation and exacerbating oxidative damage in hepatocytes [76].

### 4.2. Activates Protein Kinase C

The protein kinase C (PKC) family is recognized as the largest family of serine/threonine-specific kinases, accounting for approximately 2% of human kinases [77]. Initially discovered as an enzyme activated by proteolysis, subsequent research has revealed that various isoforms of PKC are activated upon the binding of multiple hormones to their specific receptors [78,79]. This activation is attributed to the hormones’ ability to stimulate members of the phospholipase C family upon receptor binding, leading to the production of the lipid-derived second messenger diacylglycerol (DAG), which is a primary activator of PKC [79]. Once activated, PKC translocates from the cytoplasm to the plasma membrane and various cellular organelles, where it interacts with substrates [80].

**Figure 5:**
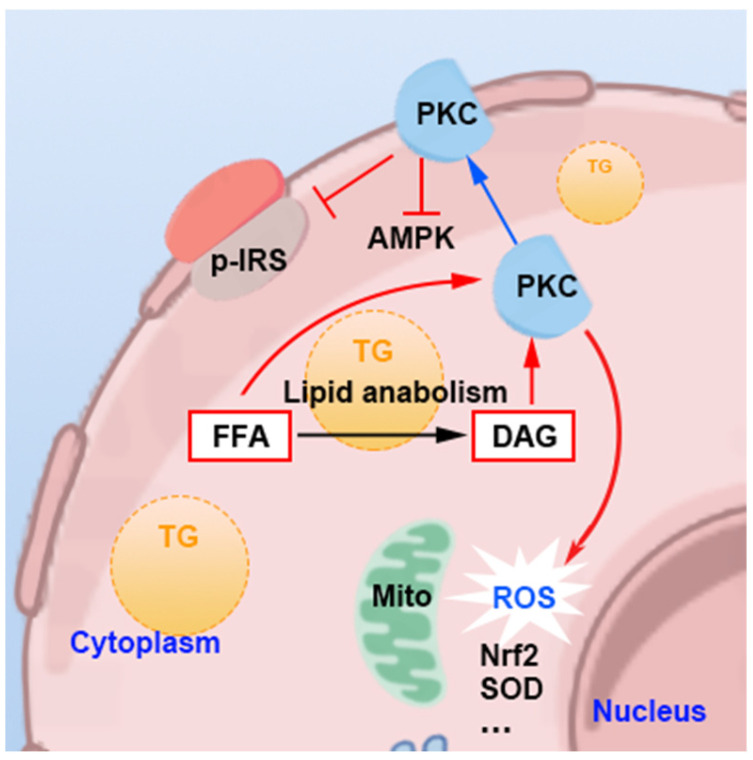
Mechanism of hepatocyte injury induced by free fatty acids via the protein kinase c pathway. p-IRS: phosphorylated insulin receptor substrate. PKC: protein kinase C. FFA: free fatty acid. DAG: diacylglycerol. Mito: mitochondria. ROS: reactive oxygen species. TG: triglyceride. Red arrows: activate; black arrows: metabolic pathway; blue arrows: translocate.

In the context of AO leading to hepatic metabolic dysfunction and lipid accumulation, PKC and its isoforms are activated through different pathways (Figure 5). They contribute to hepatocyte degeneration and exacerbate insulin resistance by modulating cellular autophagy and altering insulin receptor substrate phosphorylation [81,82]. FFAs activate PKC in the liver, thereby enhancing the exocytosis of insulin-resistant cells and opening chloride channels, which in turn intensifies insulin resistance [83].

In the synthesis and metabolism of triglycerides, diacylglycerol (DAG) exists in three stereoisomers: sn-1,2-DAG, sn-2,3-DAG, and sn-1,3-DAG [84]. Among these, sn-1,2-DAG has been proven to be the sole stereoisomer that activates the novel PKC isoforms [84]. The accumulation of sn-1,2-DAG in the liver not only activates PKC-δ, which induces ERS and participates in the regulation of MASH, but also activates PKC-ε, which mediates the phosphorylation of the insulin receptor kinase (IRK) at T1160, leading to a decrease in phosphorylation at IRK-T1162 and resulting in insulin resistance [82]. In addition to this, DAG and FFA activate PKC, leading to a decrease in autophagy capacity by inhibiting the AMPK and PI3K pathways. This results in a weakened autophagic ability in liver cells with excessive lipid accumulation, causing hepatic steatosis [81,85]. Under the conditions of DAG and OS, PKC-β is activated, and through the PKC-β/p66Shc/ROS signaling pathway, it causes mitochondrial dysfunction, increasing the production of ROS during oxidative reactions, thereby exacerbating OS [86,87].

### 4.3. Inflammation

**Figure 6:**
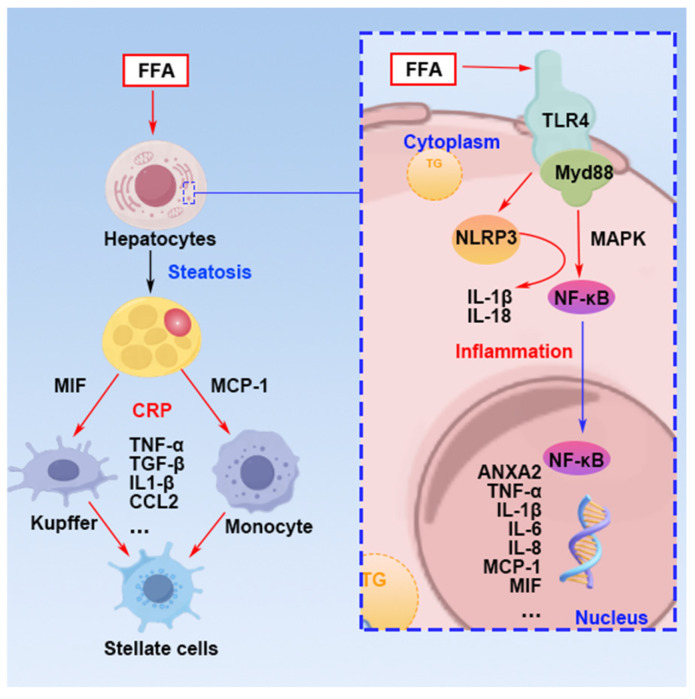
Mechanisms of excess free fatty acids cause inflammation in hepatocytes. FFA: free fatty acid. TG: triglyceride. Red arrows: activate; blue arrows: translocate.

Fatty liver is often accompanied by chronic low-grade inflammation. Inflammatory cytokines released by hepatic adipose tissue, such as tumor necrosis factor-alpha (TNF-α) and interleukin-6 (IL-6), can impair insulin signaling pathways, leading to insulin resistance. These inflammatory factors can activate intracellular stress response pathways, such as nuclear factor kappa-light-chain-enhancer of activated B cells (NF-κB), further suppressing insulin signaling (Figure 6).

Excessive lipid accumulation in liver and abdominal subcutaneous adipocytes leads to the secretion of inflammatory cytokines by adipocytes, recruiting macrophages and monocytes, thereby triggering autophagy. When adipocytes experience stress due to excessive energy intake, they secrete MCP-1 (monocyte chemoattractant protein-1) and MIF (macrophage migration inhibitory factor) [88]. Excess FFA in the liver can directly activate toll-like receptor 4 (TLR4) on the membranes of hepatocytes and macrophages, thereby inducing autophagy [89]. In hepatocytes, TLR4 mediates the enhancement of de novo ceramide synthesis and further activation of ceramide-induced protein phosphatase 2A (PP2A), which inhibits insulin signaling by suppressing Akt phosphorylation [90]. Additionally, TLR4 activates downstream NF-κB and c-Jun transcription factors, which enter the nucleus and bind to the promoter region of ANXA2, reducing autophagy by promoting ANXA2 expression, leading to steatosis [91]. Studies have found that the upregulation of ANXA2 is associated with liver steatosis, inflammation, and fibrosis; thus, hepatocytes, under the stimulation of excessive lipid accumulation and FFA, accelerate the transition to inflammation and fibrosis through ANXA2 [92]. The activation of TLR4 on Kupffer cells (liver macrophages) promotes the transformation of fatty liver to steatohepatitis through the TLR4-Myd88-NFκB pathway and, together with leukocytes, releases inflammatory cytokines (TNF-α, TGF-β, IL1-β, and CCL2) that act on hepatic stellate cells, promoting liver fibrosis [93]. Researchers have also found that in patients with NFLD, the phosphorylation of p38 is upregulated in the liver, and macrophages under high expression of p38α secrete pro-inflammatory cytokines (TNF-α, CXCL10, and IL-6) that exacerbate steatosis in hepatocytes and the transition of non-alcoholic fatty liver disease to inflammation [94].

Additionally, the overloading of fatty acid oxidation processes in the liver leading to mitochondrial dysfunction disrupts the dynamic balance of lipids within hepatocytes, resulting in fat accumulation and subsequent lipotoxicity. This is characterized by decreased mitochondrial membrane potential, increased production of ROS, enhanced translocation of cytochrome C into the nucleus, and activation of the caspase-9 apoptotic signaling pathway [95,96]. The excessive FFA mentioned earlier can induce ERS within hepatocytes. Under conditions of ERS, the NLRP3 inflammasome is activated, which, through the NF-κB pathway, triggers hepatocyte inflammation and upregulation of Chop. This, in turn, activates caspase-1, caspase-11, interleukin-1β, caspase-3, and BH3 proteins, promoting pyroptosis and apoptosis in hepatocytes [97].

### 4.4. Hormone

The accumulation of fat in the liver can lead to the release of a greater amount of adipokines (such as leptin and adiponectin) from adipose tissue, which can negatively impact insulin signaling. Both adipokines secreted by adipose tissue and hormones secreted by other tissues are involved in the regulation of lipid metabolism in the liver. Excessive free fatty acids (FFAs) due to abdominal obesity and excessive visceral fat may affect the secretion of these hormones, leading to lipid metabolism disorders and the development of insulin resistance in the liver. Adipokines primarily secreted by adipose tissue include leptin, adiponectin, and resistin [98].

Leptin stimulates fat breakdown and the release of FFAs by directly reducing the expression of FAS and increasing the expression of PPARα and FFA oxidation enzymes [99]. Studies on experimental models of non-alcoholic fatty liver disease (NAFLD) have revealed a potential dual role of leptin in NAFLD, which may have anti-lipogenic effects as well as pro-inflammatory and pro-fibrogenic actions [100]. It has been found that excessive FFAs can lead to a decrease in leptin protein and mRNA levels, suggesting that in cases of abdominal obesity, excessive FFAs may reduce leptin expression, leading to fat deposition in hepatocytes [101].

Adiponectin in the liver mainly exerts anti-lipogenic effects by increasing FFA oxidation through the AMPK and PPAR-α pathways, reducing gluconeogenesis, FFA influx, and de novo lipogenesis, while also reducing apoptosis in hepatocytes [102]. Population studies have found that high levels of FFAs and low levels of adiponectin in plasma play a key role in the mechanism by which obesity promotes insulin resistance [103]. An acute reduction of FFAs is associated with decreased adiponectin concentrations, but the long-term effects of FFA stimulation on adiponectin secretion have not been studied [104].

Resistin is involved in glucose and lipid metabolism in the liver, contributing to insulin resistance and the development of obesity, and its secretion increases with liver damage in fatty liver disease [105]. FFA deposition can exacerbate insulin resistance by increasing the release of resistin from adipocytes through OLI [106].

In addition to the impact of AO on the secretion of hormones from visceral adipose tissue, leading to abnormal lipid metabolism in the liver, hormones secreted by other organs also change their secretion under the influence of AO, causing differences in lipid metabolism.

The inhibition of FFAs can lead to enhanced responsiveness to growth hormone (GH), while an excess of FFAs may inhibit the GH signaling pathway [107]. Adult growth hormone deficiency (AGHD) is characterized by visceral obesity, dyslipidemia, premature atherosclerosis, and increased mortality, but the causal relationship between the two is not yet clear [108].

Thyroid hormones (THs) directly regulate lipogenesis, fatty acid β-oxidation, cholesterol synthesis, and the reverse cholesterol transport pathway under normal and altered thyroid hormone states [109]. Changes in the expression of thyroid hormone-related genes in adipose tissue (AT) and skeletal muscle (SM) indicate a reduced action of TH in obese tissues [110]. The inability to increase the expression of TH-related genes during periods of obesity and excess FFA supply may exacerbate lipotoxicity [110].

Testosterone and estrogen are also involved in the process of fat breakdown, but whether FFA affects the secretion of these hormones requires further research.

In summary, the occurrence of a fatty liver due to AO is often accompanied by insulin resistance. During insulin resistance, the liver’s response to insulin is reduced, leading to increased hepatic glucose production (gluconeogenesis) and further exacerbating hyperglycemia. This hyperglycemic state prompts the pancreas to secrete more insulin, creating a vicious cycle.

In conclusion, inappropriate diets are significantly associated with the development of AO, with high energy and specific nutrition intake. The excessive consumption of UPFs showed a correlation with AO risk and overweight status in the meta-analysis study. However, whether its impact on AO is greater than that on overweight in general requires further validation through randomized controlled trials and precise measurement of relevant indicators. Healthy dietary pattern interventions, such as the Mediterranean diet, along with food supplementation (e.g., milk) and nutrient supplementation (e.g., vitamin D), have been proven effective in mitigating and improving AO.

**Figure 7:**
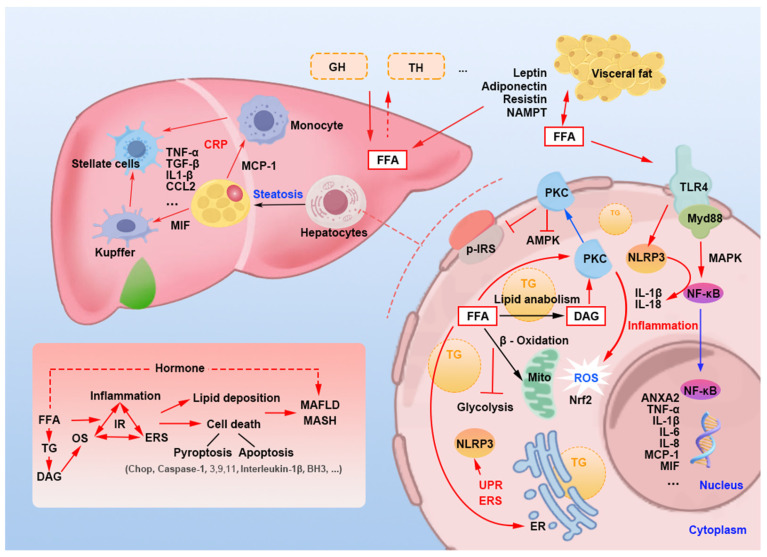
Mechanisms of excess free fatty acids exacerbating metabolic dysfunction-associated steatotic liver disease in hepatocyte. FFA: free fatty acid. TG: triglyceride. GH: growth hormone. TH: thyroid hormones. PKC: protein kinase C. p-IRS: phosphorylated insulin receptor substrate. DAG: diacylglycerol. OS: oxidative stress. Mito: mitochondria. ROS: reactive oxygen species. ER: endoplasmic reticulum. UPR: unfolded protein response. ERS: endoplasmic reticulum stress. IR: insulin resistance. MASLD: metabolic dysfunction-associated steatotic liver disease. MASH: metabolic dysfunction-associated steatohepatitis.

The primary distinction between AO and general obesity lies in the substantial accumulation of visceral fat. An unhealthy diet exacerbates this condition through gut–liver metabolism, leading to IR and inflammation activation of 11β-HSD-1, which increases endocortisol secretion and promotes visceral fat deposition. The increase in visceral fat results in elevated levels of FFAs entering the liver, where they exacerbate lipid deposition and hepatocyte death via OS, ERS, and inflammation. The metabolic products of FFAs in hepatocytes, such as diacylglycerol (DAG), further intensify OS and IR by activating PKC. Additionally, FFAs present outside the liver can potentiate lipid deposition within the liver through adipokines, GH, and TH. Through these pathways, increased visceral fat progressively exacerbates MASLD, potentially leading to its progression to MASH and fibrosis (Figure 7).

## 5. Discussion

AO and MASLD are issues that require attention across all age groups worldwide, with a particular emphasis on Asian countries. Recent population surveys in countries like the United States and China have found that the prevalence of AO among adults is higher than that of general obesity [5]. Due to differences in fat distribution between Asian and Caucasian populations, Asians tend to have more AO and a higher risk of developing MASLD at relatively lower Body Mass Index (BMI) levels. From 1990 to 2020, the highest increase in the number of people with MASLD globally shifted from China to India, and the incidence and damage associated with MASLD (DALYs) in various Asian countries are more severe compared to other nations [5].

Currently, numerous studies have focused on evaluating and predicting the risk of MASLD using various methodologies, including nomogram models and machine learning, based on population physical data and biochemical data [111,112,113]. However, these studies often lack the incorporation of diverse genetic data, environmental data, and hormonal levels. Moreover, the mechanisms by which various indicators influence MAFLD require further validation.

The relationship between AO and MASLD is closely linked, yet the magnitude of its effect on the course of MASLD still requires further validation. Studies have indicated that the primary risk factors for fatty liver disease in China include AO, general obesity, and other metabolic syndrome indicators [114]. The majority of epidemiological and pathological research has focused on the correlation and mechanistic interactions between general obesity and MASLD [115]. Compared to the subcutaneous fat accumulation in general obesity, visceral fat in AO is more likely to undergo ectopic deposition. Visceral fat produces FFAs that do not need to enter systemic circulation but instead directly enter the liver through the portal vein, disrupting hepatic lipid metabolism and affecting the normal function of other organs. Thus, we propose that AO is more harmful to MASLD than general obesity. Metabolic imbalances in the liver, in turn, lead to visceral fat deposition through inflammation and IR, causing AO; AO, in turn, exacerbates MASLD by releasing excessive FFAs. There is a complex, interrelated relationship between AO and MASLD. However, studying the effect size of AO in the formation of MASLD can provide a theoretical reference for determining whether individuals at risk of AO who have not yet developed the disease require further diagnosis of MASLD.

Due to the characteristics of visceral fat, the mechanisms of its ectopic lipid deposition mainly differ from subcutaneous fat in terms of the secretion levels of cortisol, insulin, and inflammatory factors. Hormones secreted by adipose tissue and other organs of the body have also been proven to participate in liver lipid metabolism pathways. Subsequently, we plan to explore the relationship between diet and visceral fat and further investigate the targets by which visceral fat, distinct from subcutaneous fat, causes MASLD, through research on hormones and inflammation.

## Figures and Tables

**Table 1:** Major types of dietary patterns.

Diet Pattern	Content
Mediterranean diet	High intake of olive oil and plant foods; Low-to-moderate intake of dairy products, fish, and poultry; Moderate intake of alcohol; Low intake of red meat and sweets.
Paleolithic diet	Food varieties vary by geographic location and climate; Predominantly plant- and animal-based foods; Limited dairy, salt, alcohol, sugar, grains, and processed products.
Low-fat diet	Intention is for participants to reduce dietary fat intake to ≤30% energy (≤30%E) from fat; At least partially replace the energy lost with carbohydrates (simple or complex), protein, or fruit and vegetables.
Vegetarian diet	Mainly composed of plant-based foods, with limited or complete exclusion of animal products.
Very-low-calorie ketogenic diet	Low carbohydrate content (50 g/day); 1–1.5 g of protein/kg of ideal body weight; 15–30 g of fat/day; a daily intake of about 0.5 to 0.8 kilocalories.
Ketogenic diet	Fat accounts for 70–80%, or even higher, of total calorie intake; Protein accounts for 15–20% of total calorie intake; Carbohydrates account for 5–10% of total calorie intake.
Diet high in antioxidants	Dietary composition rich in antioxidant foods: berries, vegetables, nuts and seeds, fruits, legumes, and tea.
DASH	A dietary pattern designed to help prevent and control hypertension, emphasizing fruits, vegetables, whole grains, lean proteins, and low-fat dairy.
Low-fructose diet	Strictly control fructose intake, typically limiting daily consumption to no more than 25–50 g; Increase dietary fiber intake; reduce total sugar intake, particularly added sugars.

## Supplementary Materials

The following supporting information can be downloaded at: https://www.mdpi.com/article/10.3390/nu16234208/s1.
