# Supplementary material for: Inappropriate Diet Exacerbates Metabolic Dysfunction-Associated Steatotic Liver Disease via Abdominal Obesity
Source: Nutrients. 2024 Dec 5;16(23):4208. doi: 10.3390/nu16234208 (PMC11644300; doi:10.3390/nu16234208)

Supplementary S1

1. PubMed Search Query:

((((((((("waistcircumference"[Title/Abstract]) OR ("wc"[Title/Abstract])) OR ("abdominal obesity"[Title/Abstract])) OR ("central obesity"[Title/Abstract])) OR ("ao"[Title/Abstract])) OR ("waist to height ratio"[Title/Abstract])) OR ("whr"[Title/Abstract])) OR ("waist to hip ratio"[Title/Abstract])) AND (y_10[Filter])) AND ("meta-analysis"[Filter])) AND ((((("foods"[Title/Abstract]) OR ("nutrition"[Title/Abstract])) OR ("diet"[Title/Abstract])) OR ("diet pattern"[Title/Abstract])) OR ("behaviour"[Title/Abstract]))

2. Flow chart of the number of meta-reviews identified and selected.

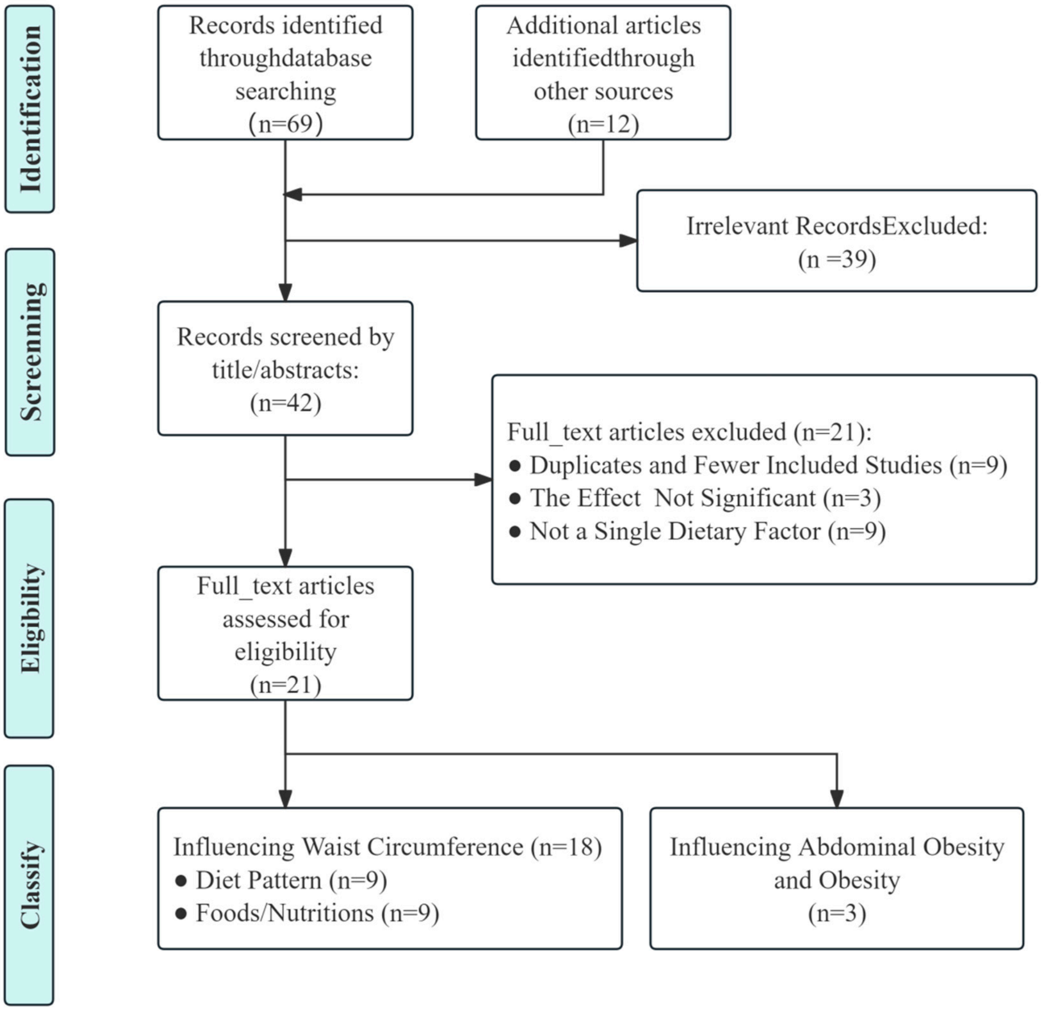

Supplement: Supplementary file 1 [file nutrients-16-04208-s001.zip › nutrients-3325382-supplementary.pdf]
